# Atrial Fibrillation Risk Management and Emerging Therapies

**DOI:** 10.3390/jcm15124612

**Published:** 2026-06-14

**Authors:** Megan Vaughan, Banveet Kaur, Nishaki K. Mehta

**Affiliations:** 1 Department of Internal Medicine, West Virginia University School of Medicine, Morgantown, WV 26506, USA; 2 Department of Cardiovascular Medicine, West Virginia University School of Medicine, 1 Medical Center Dr, Box 8500, Morgantown, WV 26506, USA; 3 Department of Cardiovascular Medicine, Corewell Health William Beaumont University Hospital, Royal Oak, MI 48073, USA

**Keywords:** atrial fibrillation, antiarrhythmic drugs, catheter ablation, anticoagulation, left atrial appendage occlusion

## Abstract

Atrial fibrillation (AF) is the most common tachyarrhythmia worldwide. Accompanying the increasing age of the general population, as well as an increase in underlying cardiovascular disease in the United States, is an explosive rise in the incidence and prevalence of this condition. We reviewed observational cohort studies, systematic reviews, meta-analyses, and randomized controlled trials (RCTs) to determine both underlying risk factors and treatment of AF, with particular focus on comorbid conditions influencing treatment success. Numerous studies have demonstrated a reciprocal relationship between maladaptive cardiac remodeling and AF, with the suggestion that aggressive management of both AF itself and resultant cardiovascular disease can lead to reversal of both conditions. Ultimately, many modifiable risk factors for AF exist, with treatment delays associated with a shift towards these conditions becoming unmodifiable. While a large area of focus for AF research has been on determining the optimal pharmacological strategy (i.e., rate versus rhythm control), results have been mixed, with emerging guidelines now pointing towards a flexible treatment strategy that allows for consideration of patient comorbid conditions, medication ease and affordability, and patient preference. Treatment of AF also includes prevention of thromboembolic events. In recent years, novel strategies for surgical or physical occlusion of the left atrial appendage (LAA) with devices such as the Watchman have arisen. Multiple large RCTs have demonstrated the safety and efficacy of these devices, but consideration must be given towards the patient’s bleeding risk, as short-term courses of blood thinners are still considered the standard of care. Finally, emerging therapies for AF include novel drug combinations, neuromodulation devices, and potentially glucagon-like peptide receptor-1 (GLP-1) agonist medications for reduction in overall metabolic disease.

## 1. Introduction

Atrial fibrillation (AF) is one of the most common arrhythmias, affecting an estimated 4.5% of (or 10.55 million) adults in the United States [1]. Of note, while older and white adults are consistently cited as those with the highest risk for AF, emerging trends suggest rising risk for younger patients, particularly in men with pre-existing hypertension or diabetes [1]. With an estimated 150,000 deaths and 450,000 hospitalizations annually attributed to AF, it is prudent that physicians recognize and appropriately treat this condition [2]. This is particularly true for younger female patients (age 25–49), and those in locations with limited healthcare access and greater burden of comorbidities, as these groups have disproportionately high rates of death compared to the general population [2]. Beyond mortality, AF is associated with increased risk of thromboembolic events such as stroke, heart failure, myocardial infarction, pulmonary emboli, and dementia [3]. Management of AF therefore must consider both treatment of the rhythm itself, and prevention of blood clots through either anticoagulants or occlusion devices. The exact treatment pathway therefore must weigh the relative risk vs. benefit of multiple medications, depending on the overall burden of comorbidities, expected lifespan, and socioeconomic status of each individual patient. This review aims to summarize the classification, risk factors, and detection of AF, as well as to provide a summary of current treatment recommendations with respect to individual patient characteristics. Lastly, we will summarize new and emerging treatment strategies.

## 2. Methods

The database utilized for this review was PubMed. Keywords utilized were “atrial + fibrillation” in addition to specific topics of interest as outlined in the text below (e.g., “atrial + fibrillation + rate + control”). Publication date was selected as “within 5 years” and article types selected were clinical trial, meta-analysis, randomized controlled trial, review, and systematic review. Evidence was weighted to favor randomized controlled trials and clinical trials, followed by meta-analyses, followed by reviews. Trials cited in current society guidelines, such as the 2024 ESC or 2023 ACC/AHA statements, were also favored.

## 3. Pathophysiology and Classification

**Figure 1:**
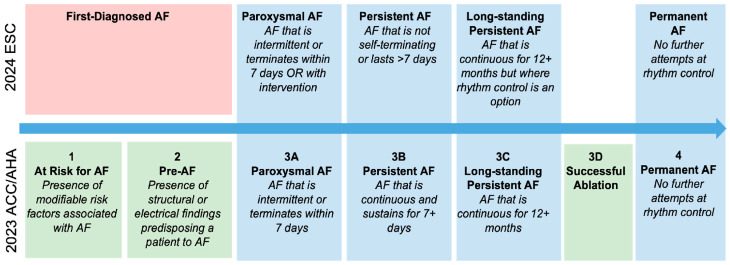
Classification of atrial fibrillation based on society guidelines. Overlap between the 2024 European Society of Cardiology (ESC, **top**) and the 2023 American College of Cardiology/American Heart Association/American College of Clinical Pharmacy/Heart Rhythm Society (**bottom**) are highlighted in blue. Classifications unique to the ESC are highlighted in red, and those unique to the ACC/AHA/ACCP/HRS are highlighted in green. AF: atrial fibrillation.

Atrial fibrillation is a type of supraventricular tachycardia caused by either rapid firing of an ectopic focus or the presence of one or more abnormal reentry circuits [4]. While these ectopic drivers of AF could arise from anywhere within the atria, typical locations include the muscle bundles surrounding the pulmonary veins, the left atrial appendage, and the ligament of Marshall [5]. AF classically presents in its paroxysmal form, with an estimated 90% of these cases driven by ectopic foci surrounding the pulmonary veins [6]. Over time, repeated episodes of AF can cause electrical, contractile, and/or structural remodeling of the atria, with these changes thought to contribute to the evolution of paroxysmal AF to persistent or even permanent AF [7]. The mechanisms behind these changes appear to be multifactorial, involving deposition of fibrotic tissue, myofibrillar loss, increased vascular permeability, and simultaneous myocardiocyte death and compensatory hypertrophy of surviving cells [8]. In addition, AF has been associated with increased inflammatory cells in the atrial myocardium, although whether systemic inflammation is a contributing factor to AF progression or merely a marker of local tissue excitability is not well understood [9]. Nonetheless, early intervention and termination of AF has been demonstrated to reverse maladaptive cardiac remodeling, thus reducing further substrate for more AF to develop [10]. Because of this natural course of the progression of AF, as well as the fact that the chronicity of the disease has implications on treatment, most older classification schemas have focused on the temporal nature of the diagnosis. The 2024 ESC guidelines, for instance, characterize AF as either first diagnosis, paroxysmal (spontaneously terminating within 7 days or with intervention), persistent (lasting greater than 7 days) or permanent (at which point no further rhythm control is pursued) [11]. In contrast, the 2023 ACC/AHA/ACCP/HRS guidelines propose a more highly stratified classification strategy which emphasizes early recognition and treatment of risk factors prior to AF development [12] (Figure 1).

## 4. Risk Factors

### 4.1. Non-Modifiable Risk Factors

**Figure 2:**
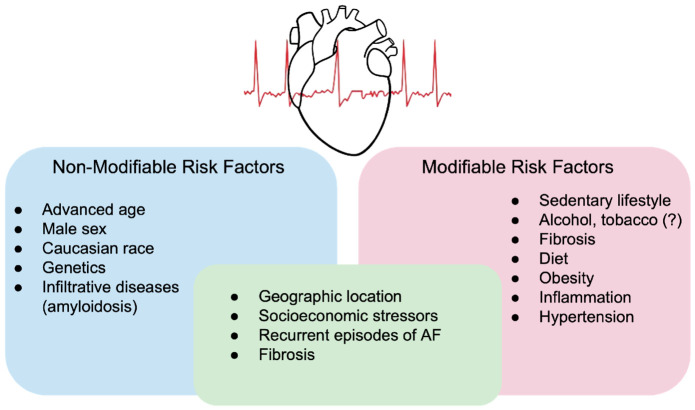
Risk factors for the development of atrial fibrillation. Includes non-modifiable risk factors (blue), modifiable risk factors (red), and those that may be either un-modifiable or modifiable over the course of a patient’s life (green). AF: atrial fibrillation.

Numerous non-modifiable risk factors for AF have been cited, such as advanced age, male sex, and Caucasian race [13]. Interestingly, while female gender and Asian ethnicity are associated with lower overall risk for AF development, they are correlated with higher overall risk of mortality due to thromboembolic events [14]. Some studies have demonstrated geographic clustering of AF diagnoses independent of underlying socioeconomic factors, although it was also noted that individuals within economically disadvantaged communities are disproportionately burdened with AF [15]. Predictably, individuals living in regions without healthcare access are also less likely to be diagnosed and/or treated for AF and thus shoulder a far greater burden of disease [16,17]. Genetics have long been implicated in the pathogenesis of AF, with studies estimating AF heritability as between 22.1 and 62% [18,19]. Since 2007, several genome-wide association studies (GWAS) have been carried out which determined 26 single nucleotide polymorphisms (SNPs) associated with AF, nine of which were associated with increased risk of stroke [20]. To date, genetic variants in the I/A/M-bands of titin, chromosomal regions related to cardiac conduction pathways [21], TNNI3 (a regulator of cardiac contraction–relaxation cycles) [22], and SYNPO2L (involved in the structural development/function of cardiomyocytes) [23] amongst many others [24] have been identified (Figure 2).

### 4.2. Modifiable Risk Factors

Modifiable risk factors for AF development have been well-described in the literature, and early recognition and treatment of these risk factors form the cornerstone of modern guidelines for AF prevention and treatment. See Figure 2.

#### 4.2.1. Sedentary Lifestyle

Physical activity has long been recommended by physicians for the prevention of cardiovascular disease, but the link between sedentary lifestyle and AF burden independent of other cardiometabolic comorbidities remains a question of interest. In one trial investigating self-reported activity levels in young Korean individuals and incident AF, the authors found no significant association between physical activity and AF diagnosis within a median of 5.6 years of follow-up [24]. A different prospective observational study of postmenopausal women followed for an average of 11.5 years did find an association between lower self-reported physical activity and AF (HR 0.9), as well as an independent association between increased body mass index (BMI) and AF (HR 1.12) [25]. One explanation for the apparent discrepancy in these studies is the observed difference in association between exercise intensity and AF in men and women, with a recent meta-analysis demonstrating a significant reduction in AF burden in both genders with moderate exercise, but an increase in AF burden in men participating in vigorous physical activity [26]. Other studies have varyingly reported either no association between high-intensity exercise and AF [27] or a significant dose-dependent relationship between daily exercise activity and AF risk [28], suggesting that physical activity may reach a certain physiological threshold, above which patients are paradoxically at increased risk for arrhythmias. Yet another study demonstrated that 24 months of high-intensity exercise resulted in positive left atrial remodeling without significant electrical remodeling, suggesting a possible mechanism by which exercise might induce AF in competitive athletes [29]. Despite this, overwhelming evidence suggests that for most patients, light to moderate activity significantly reduces AF risk, AF burden in those with established disease, and overall cardiovascular morbidity and mortality [30,31,32].

#### 4.2.2. Diet and Obesity

Multiple substances have been associated with increased risk for AF independent of obesity. Higher levels of alcohol consumption have repeatedly been shown to increase risk of AF, with one meta-analysis demonstrating that high levels of alcohol intake (variably reported as either ≥2 drinks/day in women or ≥3 drinks/day in men, >69 g alcohol/week, or >14 drinks/week) confers a significantly increased risk of AF [33]. In addition, multiple studies have reported gender stratification of this effect, with moderate intake in men also associated with increased AF, but not in women [34]. In fact, two-thirds of all hospitalizations for AF in men have increased alcohol consumption as the precipitating factor [35]. Caffeine has often been implicated as another possible precipitant for arrhythmias in general and AF specifically; however, few or no studies have demonstrated a clear link between caffeine consumption and AF [36]. In fact, the DECAF randomized controlled trial showed less recurrence of AF after successful ablation when patients were randomized to one coffee a day, compared to abstinence from caffeine [37]. Similarly, multiple studies have shown no convincing link between chocolate consumption and incident AF [38,39]. Tobacco smoking has been shown in several studies to increase AF risk, although one meta-analysis suggested that this association was stronger amongst current smokers compared to former smokers [40]. In contrast, another study demonstrated no link between tobacco use and post-operative AF in patients undergoing cardiac surgery [41]. Yet another meta-analysis showed a modest increase in AF with current tobacco use, but no reduction in risk with smoking cessation, suggesting an incompletely understood relationship between tobacco use and AF risk [42].

#### 4.2.3. Fibrosis

As discussed above, initiation of AF is classically described as originating from one or more rapidly firing ectopic foci within the left atrium. Initiation of AF can, however, also arise from one or more abnormally propagating circuits within the left atrium. Indeed, even singular atrial foci often require the development of abnormal conduction circuits to propagate abnormal firing into a sustained rhythm. This suggests that the maintenance of fibrillation is dependent on the presence of atrial myocardial cells with abnormal conduction patterns and/or abnormal repolarization characteristics. Several studies have suggested that such cells can form an “electrical scar” capable of sustaining the arrhythmia through the formation of new reentry circuits and abnormal firing patterns. These areas of scarred myocardium are typically distributed in regions of the atria that have been implicated in AF development, such as the pulmonary veins [43]. Interestingly, these regions have been demonstrated to overlap with areas of high shear wall stress within the left atrium, suggesting a possible mechanistic link between underlying precipitant conditions increasing LA wall stress (cardiomyopathy, mitral regurgitation) and the development of AF [44]. Data from the DECAAF I trial, which studied AF recurrence after catheter ablation, demonstrated that fibrosis levels correlated with recurrent arrhythmias [45,46] although a subsequent study (DECAAF II) failed to show a difference in AF recurrence with MRI-guided fibrosis ablation compared to traditional pulmonary vein isolation (PVI) [47]. The fact that AF itself also contributes to left atrial dilation, increased wall stress and scarring (and indeed, fibrosis can be used as a marker for disease progression) suggests a reciprocal feedback loop between fibrosis and AF, highlighting the importance of early intervention [48].

#### 4.2.4. Inflammation

Multiple studies have shown a link between systemic inflammation and AF risk, especially in the context of other underlying comorbidities. Data collected from patients with implantable loop recorders demonstrated a temporal and dose-responsive relationship between C-reactive protein (CRP) levels and AF occurrence [49]. This correlates well with other studies showing that pro-inflammatory states such as periodontal disease [50] and obstructive sleep apnea [51] are associated with higher rates of cardiovascular disease and AF, although in the latter study, continuous positive airway pressure (CPAP) use did not significantly decrease inflammatory markers. A meta-analysis examining studies on patients with prior stroke with or without AF demonstrated that those with AF had higher levels of high sensitivity CRP (hsCRP) that persisted longer after stroke compared to those without [52]. In the same analysis, patients with prior AF showed higher rates of recurrent adverse cardiac events but not stroke. The systemic immune-inflammation index (SSI) has also been demonstrated to be a good predictive measure of AF recurrence following ablation [53], suggesting a reciprocal relationship between AF and inflammatory states.

#### 4.2.5. Hypertension

A recent meta-analysis examining 68 studies investigating the link between hypertension and AF showed a non-linear relationship between diastolic blood pressure and AF (with a steeper risk at lower BPs), and a linear relationship with systolic blood pressure [54]. This same analysis identified increased risk even within the normal range, with pressures >180/110 mmHg associated with 1.8–2.3-fold higher risk compared to those with pressures around 90/60. An additional meta-analysis examining the correlation between circadian fluctuations in blood pressure, concomitant AF, and stroke, found that AF was an independent risk factor for “wake-up” strokes, independent of blood pressure [55]. One retrospective observational cross study found that left atrial size was positively associated with development of AF in patients with hypertension, suggesting a mechanistic link between the two [56]. Finally, the SPRINT trial studied patients with either preexisting or new onset AF to determine if intensive blood pressure control (BP < 120/80 mmHg) decreased adverse cardiac events (composite of myocardial infarction, acute coronary syndromes, stroke, heart failure, or cardiac death), but found no difference at 3 months of treatment compared to matched controls without AF, highlighting the importance of early risk factor mitigation prior to development of disease [57].

## 5. Detection

### 5.1. Subclinical

Typical presenting symptoms for patients with new onset AF are palpitations, dyspnea, fatigue, or general malaise. Up to 40% of patients, however, have subclinical or asymptomatic AF, often only being diagnosed with the arrhythmia in the context of a separate medical issue [58]. Early detection of AF is critical for prevention of progression to a permanent state, as well as development of consequent cardiovascular disease such as heart failure, cardiomyopathy, and stroke. Several studies have been carried out to identify patients at the highest risk of AF development, as well as to determine the optimal screening strategy to balance early detection and overscreening. The Dx-AF study compared a novel implantable cardioverter defibrillator (ICD) device with an atrial sensing lead (VDD-ICD) with a traditional single-chamber ICD and measured time to detection of first AF episode [59]. The authors found that the VDD-ICD device significantly increased detection of AF (HR 2.36), although the results were not significant due to limited sample size (*p* = 0.15). A review paper examining eight clinical trials aimed at detecting silent AF with implanted devices found a high burden of silent AF across all studies, particularly in those with prior episodes of AF; although, up to 34.7% of patients with silent AF had never been previously diagnosed with an arrhythmia [60]. Several of these studies found that the presence of silent AF events directly correlated with overall mortality [61] and stroke [62,63], although other studies found few patients had recorded subclinical AF events within a month of their stroke [64], suggesting that silent AF may impact overall embolic event risk through indirect mechanisms such as cumulative remodeling of the left atrium.

### 5.2. Perioperative

AF is frequently detected for the first time in the perioperative period, with rates for new-onset postoperative AF for some surgical subtypes (such as cardiac surgery) estimated to be as high as 60% [65]. While common, it is not without risk, as some studies have shown an increase in perioperative mortality (OR 1.92), perioperative stroke (OR 2.17), perioperative MI (OR 1.28), long-term mortality (IRR 1.54), long-term stroke (IRR 1.33) and longstanding persistent AF (IRR 4.73) [65]. Other studies have demonstrated similar findings for postoperative burn patients [66], CABG [67], and emergency abdominal surgery [68]. To manage these risks, various surgical societies such as the Society of Thoracic Surgeons have created specific guidelines for management of new-onset AF in the setting of cardiac surgeries [69].

## 6. Management

### 6.1. Rate vs. Rhythm Control

Numerous studies have been conducted to examine whether a rate control or rhythm control strategy for management of AF has superior outcomes. Despite this, no clear consensus has been reached. Part of this can be explained by differences in primary outcomes across clinical trials, rather than directly contradictory evidence. Restoration of sinus rhythm, reduction in AF burden, reduction in hospitalizations, reduction in heart failure, and improvement in quality-of-life metrics are all equally important, but fundamentally different, outcomes. Due to the underlying goal of rate and rhythm control strategies being different (i.e., maintenance of ventricular rate despite underlying heart rhythm vs. reduction in AF rhythm itself), few trials have been conducted comparing these strategies in terms of efficacy of sinus rhythm restoration. Rather, most clinical trials have focused on reductions in the sequelae of unchecked AF. For example, one meta-analysis examining 18 studies comparing rate control (beta-blocker, digitalis, or calcium channel blocker) to rhythm control (antiarrhythmic medications, catheter ablation, electrical cardioversion) found a reduction in cardiovascular mortality and stroke with rhythm control [70]. In another review examining patients with persistent or recurrent AF, rate control was found to be at least noninferior to rhythm control in terms of cardiovascular mortality [71]. Many large clinical trials have also shown comparable efficacy of rate control vs. antiarrhythmics/cardioversion. For example, AFFIRM showed noninferiority with respect to survival. In the HOT CAFE trial, the same was found for all-cause mortality, thromboembolic events, or major bleeding [72,73]. Other trials have opted for a patient-centered approach when choosing primary outcomes. For instance, the PIAF trial showed no difference in overall patient quality of life when comparing use of diltiazem and amiodarone but noted that exercise performance was improved in the amiodarone group [74]. In the STAF trial, 200 patients with persistent AF were randomized to receive rate vs. rhythm control; no significant differences were found in composite of death, cardiopulmonary arrest, CVA, or embolic events, though patients in the rhythm control group had higher rates of hospitalization [75]. Underlying comorbidities may also play a role in the efficacy of each strategy. For instance, one study found that in patients with underlying heart failure with reduced ejection fraction (HFrEF, EF < 40%), a rhythm control strategy decreased rate of heart failure progression over a median follow-up of 4 years [76], while another study examining patients with congestive heart failure (EF < 35%) found the opposite [77]. An additional consideration when choosing rate vs. rhythm control is the duration of AF. The EAST-AFNET4 trial showed that early rhythm control (AF diagnosed within 12 months prior to trial initiation) in patients with a CHA_2_DS_2_-VASc score ≥ 4 reduced the composite outcome of cardiovascular death, stroke, hospitalization for worsening heart failure, or ACS when compared to usual care (initial rate control strategy with rhythm control reserved as rescue therapy). The decision to pursue a rate vs. rhythm control strategy must therefore take into consideration multiple factors such as duration of AF, whether it is new or recurrent AF, underlying comorbidities, heart failure phenotype, age of the patient, medication safety profile, patient preference, and feasibility of long-term follow-up.

### 6.2. Management of Acute AF

**Figure 3:**
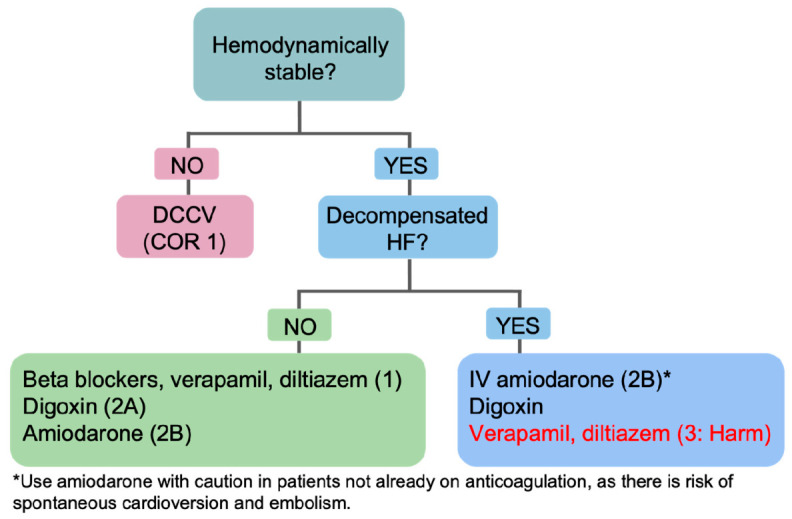
Algorithm for treatment of acute atrial fibrillation. COR shown are based on 2023 ACC/AHA/ACCP/HRS guidelines. DCCV = direct-current cardioversion. Please note that this is a simplified algorithm for educational purposes, and should not replace individualized clinical decision-making.

Management of acutely presenting AF largely depends on two factors: the stability of the patient, and whether they are adequately anticoagulated or not. For patients who present with hemodynamic instability, which is defined as hypotension (especially requiring pressors), signs of end-organ damage, severe tachycardia (HR > 140), suspected or confirmed myocardial infarction or severe illness, or evidence of pre-excitation syndrome on ECG, the 2023 ACC/AHA/ACCP/HRS guidelines have a class 1 recommendation for direct current cardioversion (DCCV). For stable patients who have been in AF less than 24 h (based on the 2024 ESC guidelines) or 48 h (based on the 2023 ACC/AHA guidelines), cardioversion may also be performed. For stable patients in AF ≥ 24–48 h, more consideration must be made regarding whether the patient has been on anticoagulation for ≥3 weeks, as cardioversion to sinus rhythm risks a thromboembolic stroke. In these cases, physicians may opt to perform a transesophageal echocardiogram (TEE) prior to cardioversion, or to pursue a pharmacologic strategy as either permanent therapy, or until the patient has been adequately anticoagulated for cardioversion. Pharmacologic strategies for patients who are stable and not in acute decompensated HF include beta blockers (class 1 recommendation), calcium channel blockers (CCB, class 1), or digoxin (class 2a) [78]. Amiodarone may be used with caution, as patients are at higher risk of spontaneous cardioversion. For patients presenting in acute heart failure, amiodarone carries a 2b recommendation. CCBs in these patients are to be avoided, as diltiazem has been demonstrated to significantly increase HF progression [79] (Figure 3).

### 6.3. Long-Term Management of AF

Long-term treatment of AF relies less on initial selection of monotherapy and more on flexibility in adapting treatment strategy based on patient comorbidities, age, functional status, and preferences. As mentioned previously, the AFFIRM trial demonstrated no difference in efficacy between rate and rhythm control strategies, although a post hoc analysis did demonstrate that beta-blockers achieved ventricular rate goals in 59 percent of people, suggesting that they may be reasonable first-line agents [80]. Still, many patients do not respond adequately to the initial pharmacological agent and will require a trial of several classes of medications to achieve either a sinus rhythm or ventricular rate below the goal. Nevertheless, numerous studies have examined predictive factors for initial agent success. Factors favoring rate control are older (age ≥ 75)/asymptomatic patients, those with a large left atrium, those with no prior history of heart failure, those with an AF duration > 2 years, and patients at higher risk of syncope [81]. Alternatively, rhythm control may be favorable for more active patients in whom preservation of exercise capacity is a priority [82], those with an AF duration < 1 year [83], small/normal left atrial size, HFpEF (especially if symptomatic), EF < 55% (especially if asymptomatic), presence of ICD, valvular disease, structural heart disease, high symptom burden, and age ≤ 75 years [84].

#### 6.3.1. Rate Control Agents

**Figure 4:**
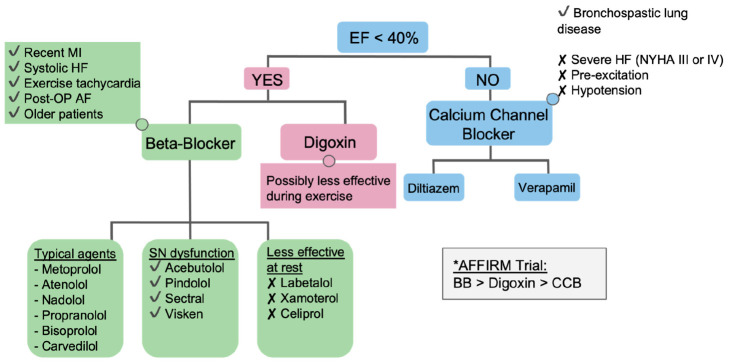
Algorithm for selection of rate control agent in the long-term treatment of atrial fibrillation. Check marks indicate agents that are indicated, while x’s indicate those that are contraindicated. For relevant supporting studies, please see the accompanying text. MI = myocardial infarction, HF = heart failure, SN = sinus node. * Based on a post hoc analysis of the AFFIRM trial, demonstrating efficacy of various monotherapy agents in achieving both rest and exercise HR < 80 at average follow-up 3.5 ± 1.3 years [85]. Please note that this is a simplified algorithm for educational purposes, and should not replace individualized clinical decision-making.

A post hoc analysis of the AFFIRM trial examined over 2000 patients to compare the effectiveness of different rate control agents at achieving either resting heart rate of ≤80, exertional heart rate of ≤110 or average heart rate of ≤100 [85]. The overall effectiveness was greatest for beta-blockers (59%), followed by digoxin (58%) and then CCBs (38%). The choice of agent must also take into consideration the patient’s underlying comorbidities. For instance, CCBs are considered first-line for patients with underlying bronchospastic lung disease but are contraindicated in those with severe HF (NYHA III or IV), pre-excitation syndromes, or hypotension [86]. Amongst the CCBs, diltiazem and verapamil have demonstrated equal efficacy [87]. For patients with severe HF, either digoxin or beta-blockers may be used. Of note, digoxin might be less effective at controlling heart rate during exercise, although this effect may be mitigated by the addition of a small dose of an alternative agent [88]. Beta-blockers (BB) are typically considered the first-line choice for patients with recent MI, severe systolic HF, exercise tachycardia, post-operative AF, and older patients. Choice of a specific beta-blocker is largely dictated by individual physician preference and hospital availability. Amongst the typical agents used, metoprolol, atenolol, nadolol, propranolol, bisoprolol, and carvedilol have similar efficacy. For patients with sinus node dysfunction and/or tachy-brady syndrome, a BB agent with intrinsic sympathomimetic activity (acebutolol, pindolol, sectral, visken) may be useful. Recently, the FDA has approved use of the ultra-short-acting cardio-selective beta-blocker landiolol, which was previously available only in Japan and the European Union; early RCTs have shown promise for this medication in the treatment of perioperative and sepsis-related AF [89] (Figure 4).

#### 6.3.2. Rhythm Control Agents

**Figure 5:**
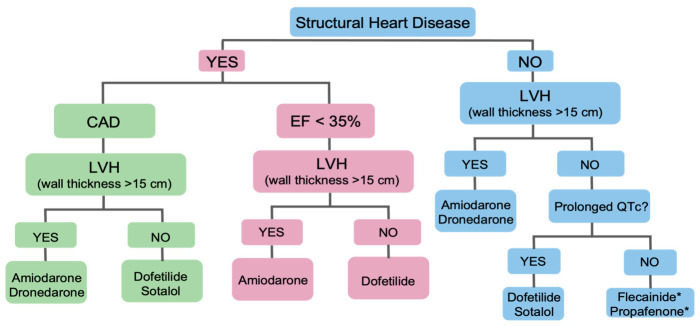
Algorithm for selection of rhythm control agent in the long-term treatment of atrial fibrillation. * Should be combined with AV nodal blocking agents. Please note that this is a simplified algorithm for educational purposes, and should not replace individualized clinical decision-making. CAD: coronary artery disease; LVH: left ventricular hypertrophy; EF: ejection fraction; QTc: corrected QT interval.

Selection of an antiarrhythmic medication largely depends on the presence of underlying structural heart disease (CAD or HF). For patients < 70 years old without structural heart disease, current guidelines recommend the use of flecainide or propafenone as first-line agents due to their favorable side-effect profile, with second-line agents being dofetilide, sotalol, amiodarone, or dronedarone, with the latter two being preferred for patients with evidence of left ventricular hypertrophy (LVH, wall thickness > 15 mm) [90]. For patients with underlying CAD, amiodarone/dronedarone are preferred for those with LVH, while dofetilide/sotalol are used for those without LVH. Similarly, for patients with HF (EF < 35%), amiodarone is the agent of choice for patients with LVH and dofetilide for those without. Numerous studies have been performed to determine if the mechanism of AF (i.e., triggered activity, independently firing foci, or reentry circuits) influences efficacy of a particular agent. Results from CAST, SWORD, and PALLAS have demonstrated clinical harm when this strategy is used, indicating that underlying comorbidities may be more important in antiarrhythmic selection than AF mechanism [91] (Figure 5).

#### 6.3.3. Ablation

Catheter ablation should generally be considered as either a primary therapy or rescue treatment for any patients in whom a rhythm control strategy is being considered. The 2023 ACC/AHA/ACCP/HRS Guidelines have a COR 1 recommendation for catheter ablation in patients with symptomatic HF in which antiarrhythmic drugs have been ineffective, contraindicated or not tolerated, as well as in younger patients with symptomatic paroxysmal AF (especially those with high AF burden). Multiple recent studies have demonstrated that ablation is more effective than antiarrhythmic medications for both paroxysmal and persistent AF, particularly when used early in the diagnosis [92,93,94]. In the ATTEST trial, catheter ablation was found to reduce progression from paroxysmal to permanent AF [95]. Most trials have examined the use of catheter ablation in younger patients (<70), because these patients are more likely to be selected for a rhythm-control strategy, and the fact that AF burden in these patients is expected to have more time to cause maladaptive cardiac remodeling. Several studies have, however, demonstrated benefit for an early ablative strategy for elderly patients [96,97]. In patients presenting with newly diagnosed AF, it may be reasonable to start with an antiarrhythmic medication, particularly in older patients with a shorter life expectancy and therefore lower likelihood of developing significant side effect burden from these medications. Following failure of an antiarrhythmic medication, the STOP-AF trial demonstrated that using catheter ablation as a rescue strategy had a 70% success rate compared to 7% for patients switched to a different drug [98]. On the other hand, the CABANA trial showed that patients switched from an antiarrhythmic medication to catheter ablation had a nearly 50% reduction in recurrent AF compared to those who continued their medication, but this did not impact the primary composite endpoint of death, stroke, major bleeding, or cardiac arrest [99]; of note, while the primary intention-to-treat endpoint for this trial was not significantly reduced, AF recurrence and cardiovascular hospitalizations were lower for patients switched to ablation, with trial interpretation influenced by crossover. Catheter ablation should therefore be considered for patients in whom AF causes significant symptom burden or is likely to become symptomatic, rather than patients who are adequately controlled on an antiarrhythmic medication. For younger patients (especially <60 years old) with relatively few comorbidities, catheter ablation as a primary strategy is more suitable [100,101].

##### Types of Ablation

Traditionally, ablation procedures for AF consisted of using thermal energy to either isolate the pulmonary veins, or to create a network of scar tissue to prevent propagation of abnormal circuits (the MAZE procedure). Radiofrequency (RF) is the most studied thermal ablation technique and can be combined with high-density electroanatomic mapping to perform both pulmonary vein isolation (PVI) and extrapulmonary ablative procedures. Cryoballoon subsequently emerged as a second thermal technique; while it is of similar efficacy as RF, technical challenges in performing this procedure make it most suitable for PVI-only approaches [102]. More recently, non-thermal techniques have been introduced, including pulsed field ablation (PFA), which uses electroporation to selectively induce cell death in cardiac myocytes without significant increase in tissue temperature. Recent studies have demonstrated similar efficacy of PFA in obtaining complete pulmonary vein isolation with larger antral lesions compared to cryoballoon [103]. Some limited trials have also demonstrated superiority of PFA in maintaining sinus rhythm as compared to thermal techniques [104]. Indeed, the ADVENT trial demonstrated significantly less AF burden at 12 months following PFA as compared to thermal ablation [105]. Recent advancements in ablative techniques include the introduction of high-power short-duration (HPSD) and very-high-power short-duration (vHPSD) radiofrequency ablation (RF), which utilize a novel catheter tip capable of real-time assessment of tip temperature to perform safer and more effective procedures. These techniques have been demonstrated to be non-inferior when compared to traditional RF in terms of significant reduction in AF, AF recurrence at follow-up, and safety (risk of stroke, tamponade) [106]. Limited data exists comparing these techniques in terms of patient experience, though one recent study did examine patient quality of life (QoL) scores for RF, cryoballoon, HSPD, and vHPSD. The authors found that PFA patients reported the greatest improvement in QoL, largely driven by faster activity resumption and symptom relief [107]. Optimal ablation technique also depends on careful consideration of individual patient anatomy. The presence of anomalous pulmonary venous drainage, for instance, might favor a specific catheter approach [108]. Ultimately, the decision of which ablation approach to attempt depends on multiple factors, such as AF type, left atrial anatomy, patient comorbidities, operator training, prior ablative procedure(s) attempted, recurrence risk, and patient preference. Future studies are needed to more directly compare newer ablative strategies with regard to these considerations.

#### 6.3.4. Anticoagulation vs. Occlusion Devices

Multiple societies, including the ACC/AHA/ACCP/HRS and CHEST recommend that any patient with nonvalvular AF should be started on a direct oral anticoagulant (DOAC) based on risk stratification with the CHA_2_DS_2_-VASc and HAS-BLED scoring system [109]. For patients with zero non-sex CHA_2_DS_2_-VASc risk factors no DOAC is needed, while male patients with at least one risk factor or female patients with two risk factors should be started on DOAC [110]. Of note, the 2024 ESC guidelines have proposed to adopt a gender-neutral strategy, given that female gender is an independent risk factor for AF, as well as the potential for gender-inclusive algorithms to misdiagnose patients identifying as non-binary, transgender, or those actively taking hormone-based therapies. The ESC therefore recommends DOAC for patients with CHA_2_DS_2_-VASc of 2 or greater, with consideration for those with CHA_2_DS_2_-VASc of 1, irrespective of gender or birth sex. Anticoagulant therapy is vital for prevention of stroke in patients with paroxysmal AF, as well as patients who are converted to sinus rhythm, as both electrical and pharmacological cardioversion are associated with temporary myocardial stunning and increased embolism risk [111,112]. For patients with subclinical AF, the NOAH-AFNET-6 and ARTESiA trials demonstrated that anticoagulation with DOAC was superior to both placebo and aspirin for stroke prevention in very high-risk patients (CHA_2_DS_2_-VASc > 4) [113]. Patients started on DOACs should be continuously evaluated for the ongoing need for these medications, particularly in the context of elderly patients, those at high bleeding risk (based on HAS-BLED score), and those at a higher risk of falls [114]. An emerging anticoagulation class is the factor XI (FXI) inhibitors, which include abelacimab, asundexian, and melvexian. The phase 2 AZALEA-TIMI 71 trial was prematurely terminated after it demonstrated significant reduction in incidence of major bleeding events with abelacimab compared to rivaroxaban. In contrast, the phase 3 OCEANIC-AF study comparing asundexian and apixaban was stopped due to inferiority of the FXI in preventing thromboembolic events. Future trials, such as LILAC-TIMI 76 and LIBREXIA-AF, are required to confirm the efficacy of these agents. On the other hand, patients at higher bleeding risk may be considered for placement of a left atrial appendage occlusive (LAAO) device, which is thought to mitigate stroke risk through elimination of the portion of the left atrium most implicated in clot formation. Patients with either relative or absolute contraindications to DOAC therapy, such as prior intracranial bleed, may be considered for LAAO [115]. Currently, two such devices exist; the Watchman device (Boston Scientific, Boston, MA, USA), and the Amplatzer Amulet device (Abbott, Chicago, IL, USA). Randomized controlled trials examined both the efficacy and safety of these devices. The PROTECT-AF trial compared patients with nonvalvular AF and CHA_2_DS_2_-VASc score ≥ 1 assigned to either the Watchman device or dose-adjusted warfarin and found that the probability of non-inferiority of the device was more than 99.9% (RR 0.62), although the primary safety endpoints (major bleeding, pericardial effusion, device embolization, or procedure-related stroke) were also significantly higher (RR 1.69) [116,117]. In the PREVAIL trial, patients were again randomized to either Watchman device for warfarin, although inclusion criteria were patients with nonvalvular AF and CHA_2_DS_2_-VASc score ≥ 2. Of the three primary endpoints (composite hemorrhagic stroke/ischemic stroke/systemic embolism/sudden cardiac death, ischemic stroke/systemic embolism with the first 7 days after device placement excluded, or composite of all-cause death/ischemic stroke/systemic embolism/device-related complications requiring intervention), only the second endpoint (stroke or embolism >7 days post-randomization) achieved non-inferiority (risk difference 0.0053) [118]. A recent systematic review and meta-analysis, which included 10 studies comparing LAAO to DOAC, found that the occlusive device was associated with significantly lower all-cause mortality (HR 0.63) and cardiovascular mortality (HR 0.56), and exhibited a trend towards lower stroke/TIA that was not significant [119]. Still, significant risks with LAAO device placement exist, most notably device-related thrombotic events. Currently, few guidelines exist on the exact regimen and length of treatment with either DOAC or antiplatelet agents in the months following LAAO placement [120,121]. LAAO devices may therefore be appropriate for patients who are not candidates for either DOAC or warfarin long-term, but in whom short-term treatment may be tolerated [122]. In patients undergoing another major cardiovascular surgery (such as CABG), surgical ligation of the LAA has been demonstrated to reduce stroke risk by 29%, although no benefit was found in a subgroup analysis that included patients without preoperative AF [123]. Again, recommendations on blood thinners in the immediate post-operative period are mixed, and patients should be advised that a short-term period of anticoagulation may be required to minimize risk of stroke immediately following the procedure. Finally, it has long been a matter of debate whether anticoagulation should be discontinued after successful AF ablation. A recent meta-analysis examining this question found that relative risk of major bleeding events vs. thromboembolic events differed between patients depending on their CHA_2_DS_2_-VASc score (CHA_2_DS_2_-VASc ≤ 1: HR 1.51 (bleeding) vs. 0.86 (embolism) with continued DOAC, CHA_2_DS_2_-VASc ≥ 3: HR 1.05 (bleeding) vs. 0.61 (embolism) with continued DOAC), despite apparently successful restoration of sinus rhythm in both groups [124]. The decision to discontinue anticoagulation after ablation should therefore not be based only on apparent rhythm control or procedural success but should remain guided by CHA_2_DS_2_-VASc score, HAS-BLED score, clinical impression of embolic vs. bleeding risk, AF recurrence monitoring, patient frailty, and shared decision-making.

#### 6.3.5. Clinical Evaluation

All patients with newly diagnosed AF should undergo a comprehensive clinical evaluation, including a transthoracic echocardiogram to evaluate for chamber size and function, valve pathology, pressures, and strain patterns [125]. Left atrial size should be evaluated as this is predictive of therapy success (especially cardioversion and ablation) [126]. Basic laboratory testing with complete blood count, basic metabolic panel, and thyroid function testing should also be performed, both to determine underlying precipitants of AF and for the purposes of therapeutic decision-making. Baseline ECG should be performed, both during AF and after conversion to normal sinus rhythm if achieved, to identify underlying abnormalities and/or arrhythmias contributing to AF progression. AF in and of itself does not necessarily warrant an ischemic evaluation, as studies examining asymptomatic patients with AF found that the yield to detect ischemia was only 0.4% [127]. Routine evaluation for coronary artery disease or PE is therefore not recommended in asymptomatic AF patients.

## 7. Unique Challenges with Comorbid Conditions

### 7.1. Heart Failure with Preserved Ejection Fraction 6.2 Cardiomyopathies

Heart failure with preserved ejection fraction (HFpEF) is defined as a clinical syndrome with symptoms/features of heart failure with an ejection fraction of ≥45%. It is classically associated with older, female patients with a high prevalence of underlying comorbidities such as obesity, hypertension, diabetes, and chronic kidney disease [128]. HFpEF also typically has evidence of left ventricular diastolic dysfunction, pulmonary hypertension, and/or increased myocardial wall stress, all of which can be both caused by and precipitate AF [129]. Persistent AF has been associated with reactive fibrosis, a process by which collagen is deposited between cardiac muscle bands, and which leads to the diastolic dysfunction associated with HFpEF [130]. Some of these changes may be reversible with AF therapy, with one study showing resolution of HFpEF in 50% of patients within 6 months of catheter ablation compared to <10% of those treated medically [131]. Interestingly, a post hoc analysis of the AFFIRM trial showed that individuals randomized to a rhythm-control strategy had a 32% relative risk reduction in new HF diagnosis and cardiovascular death compared to rate control [132]. Furthermore, HF emergence directly correlated with the degree of rhythm control achieved, with a relative risk reduction of 69% for patients with <25% of follow-up ECGs demonstrating AF compared to those with >25%, suggesting that residual AF burden contributes to HFpEF development [133]. Superiority of rhythm control versus rate control was also demonstrated in a meta-analysis of five studies directly comparing all-cause mortality in patients with HFpEF [134]. All these studies suggest that, in addition to standard therapy for HFpEF, AF should be aggressively managed, with consideration given to a rhythm control strategy for those with clinical symptoms of HF.

### 7.2. Cardiomyopathies

Cardiomyopathies are a diverse group of disorders involving the heart musculature that result in structural, electrical, and functional abnormalities and which can result in valvular abnormalities, heart failure, and overall decline in patient functional status. Cardiomyopathies may result from long-standing AF and therefore represent disease progression or may be due genetic conditions such as familial hypertrophic cardiomyopathy (HCM) which can themselves be drivers of AF [135]. Patients with HCM and AF are managed similarly to the general population, with several notable exceptions. Because rapid ventricular rate can contribute to LVOT obstruction, a more aggressive approach to restoration and maintenance of sinus rhythm is required [136]. In asymptomatic or minimally symptomatic patients, initial management with beta-blockers or calcium channel blockers can be attempted. Digoxin is contraindicated in these patients, as its positive inotropic effect can be detrimental for HCM patients, who are dependent on LV pre-load [137]. Although there are no trials examining the efficacy of rate vs. rhythm control in this population, guidelines currently favor the use of antiarrhythmic medications in HOCM [138,139]. Similarly, a rhythm control strategy has shown to be favorable in several clinical trials examining AF-mediated cardiomyopathy [140], with ablation in particular showing favorable outcomes for this population [141]. Regardless of etiology, the most important factors in management of concurrent AF and cardiomyopathy are early intervention, management of underlying comorbid conditions, and lifestyle interventions to improve overall cardiovascular health.

### 7.3. Chronic Kidney Disease

A post hoc analysis of the GUSTO-III trial, which examined patients with CKD and newly diagnosed AF following MI, found no significant difference in the percentage of patients who achieved sinus rhythm by hospital discharge with a rate control (76%) versus rhythm control (80%) strategy (*p* = 0.2) [142]. Multiple studies have examined the impact of an early rhythm control (ERC) strategy in patients with renal disease. In a secondary analysis of the EAST-AFNET4 trial, ERC was associated with a reduced primary outcome (composite cardiovascular death, stroke, hospitalization for HF, and ACS) in both patients with and without CKD [143]. Another study found a reduction in their primary endpoint (composite cardiovascular death, ischemic stroke, HF hospitalization, and MI) for patients with CKD 3 to 4 started on ERC compared to rate control (HR 0.85), but no difference in patients with ESRD (HR 0.97), although the authors noted that poor adherence to ERC could have played a major role [144]. Multiple studies have also examined the efficacy of various antithrombotic medications for the management of AF with underlying CKD. A meta-analysis of 8 RCTs and 46 observational studies found that DOACs were superior to warfarin in preventing embolic events in this population (HR 0.86) and in reducing bleeding events (HR 0.81) [145]. A separate post hoc analysis of the VALKYRIE, AXADIA, and RENAL-AF trials found that in patients with moderate CKD (CrCl 30–50 mL/min), dabigatran and apixaban were associated with lower rates of systemic embolism and stroke, and apixaban/edoxaban were associated with lower rates of bleeding, compared with warfarin [146]. Similar findings were demonstrated for patients with advanced CKD (non-dialysis-dependent stage 4 or 5) [147].

### 7.4. Malignancies

Patients with active malignancy have an increased risk for the development of AF, with one meta-analysis of observational studies demonstrating a roughly 47% higher rate compared to patients without cancer [148]. During active cancer treatment, AF may occur with a much higher rate, although only Bruton tyrosine kinase inhibitors have a high level of evidence, with some studies reporting as high as 3–4-fold higher risk for ibrutinib specifically [149]. Beyond side effects of chemotherapeutics, malignancy itself increases the risk of AF through both overlapping risk factors for both conditions (advanced age, poor physical conditioning) and through the general proinflammatory state of cancer itself [150]. Proposed mechanisms behind the link between chronic inflammation and AF include imbalance in sympathetic and parasympathetic nerve activity, abnormal strengthening of noncholinergic vagal nerve signals, and general instability of the sympathetic nervous system [151]. Active malignancy is also associated with increased risk for thromboembolic events, independently of the risk posed by concomitant AF. Current guidelines recommend optimizing control of AF while prioritizing the continuity of cancer therapy. Drug–drug interactions between oncological therapies and medications for the treatment of both AF itself and thromboembolism risk are common and require careful management by a multidisciplinary team that includes Oncology and Cardiology. For certain chemotherapeutics, such as the BTK inhibitor ibrutinib, CYP3A-inhibiting medications (such as amiodarone, dronedarone, verapamil, or diltiazem) are associated with increased drug levels and are therefore generally avoided [152]. In patients taking ibrutinib, a rate control strategy is therefore attempted first, highlighting the need for consideration of both the underlying malignancy and treatment strategy in choosing an appropriate AF treatment plan. No large RCTs are yet available that compare warfarin and DOACs for thromboembolism prevention in patients with AF and cancer, although a post hoc analysis of patients with cancer and AF from the ROCKET AF (rivaroxaban versus warfarin) study found a lower risk of stroke or systemic embolism (RR 0.65) and bleeding (RR 0.68) with DOACs. While further studies are needed to determine the interactions between chemotherapeutics and various blood thinners, a fair approach may be to treat these patients similarly to the general population, with the caveat that active malignancy must factor into the calculation of their overall bleeding/thrombotic risk.

### 7.5. Pregnancy

Pregnancy and the post-partum state have long been recognized as significant risk factors for thromboembolic disease due to increased estrogen levels. In fact, women without underlying clotting disorders may be at up to five times higher risk for developing blood clots during pregnancy. Incidence of AF in pregnancy increases with maternal age and represents an independent risk factor for blood clot formation. Treatment of AF in pregnant patients also presents a unique challenge due to the potential maternal and fetal risks from antiarrhythmic and antithrombotic medications. Current recommendations for acute management of AF in pregnant patients are beta-blockers and digoxin as first-line agents, with second-line choices including flecainide, propafenone and sotalol. Amiodarone is generally contraindicated. Alternatively, direct cardioversion is considered effective and safe during pregnancy, given that the patient is anticoagulated prior. Anticoagulation in pregnant patients is dependent upon the stage of pregnancy. Low molecular weight heparin is typically the agent of choice for all but the final weeks of pregnancy, due to its ease of administration and the fact that it does not cross the placenta. Warfarin and other Vitamin K antagonists are generally avoided, as they freely cross the placenta and are teratogenic. For patients at exceptionally high risk of thromboembolism (for instance, those with mechanical heart valve), heparin can be used during the first trimester when teratogenic risk is highest, followed by resumption of warfarin for the second trimester onwards. Direct thrombin inhibitors may be considered for patients with contraindications to heparin, although they are less well studied in pregnancy and generally not considered first-line. DOACs should not be used in pregnancy due to increased reproductive and teratogenic risks in animal studies and insufficient data from human studies.

## 8. Emerging Therapies and Future Directions

Novel therapies for the management of AF include antiarrhythmic agents with selective affinity to ion channels involved in atrial repolarization. Specifically, ultra rapid delayed rectifier (IKUR) blocking agents, atrial specific sodium channel blockers, muscarinic (M2) receptor blockers, and five-HT4 receptor blockers [153]. Additional trials have examined the use of angiotensin-converting enzyme inhibitors (ACEIs) or angiotensin receptor blockers (ARBs) to prevent AF recurrence following cardioversion [154]. The combination of anti-arrhythmic medications with these non-anti-arrhythmic agents (ACEIs, ARBs, statins, omega 3-PUFAs) have also demonstrated greater efficacy in maintaining sinus rhythm following cardioversion compared to anti-arrhythmic therapy alone [155]. Future studies are needed to determine whether these combination therapies work to prevent aberrant electrical signaling pathways, detrimental physical and electrical remodeling, or both. More research is also needed in patients with AF in the absence of other comorbidities (such as hypertension or heart failure) that would typically warrant these non-anti-arrhythmic therapies.

Other emerging strategies involve non-invasive and percutaneous surgical interventions for autonomic modulation. Examples include tragus stimulation, renal denervation, cardiac afferent denervation, alcohol injection into the vein of Marshall, baroreceptor activation therapy and endocardial ganglionated plexi ablation [156]. Cardiac afferent denervation has been shown to be efficacious in management of both AF [157] and ventricular arrhythmias [158]. Additional surgical approaches include magnetic-based circular mapping catheters for more precise pulmonary vein isolation [159] and complex atrial electrical mapping [160]. Despite success with many of these procedures, the underlying mechanistic pathways connecting the autonomic nervous system and AF remain poorly understood. Future research is needed to determine which patients would most benefit from a surgical approach to AF management, versus medical therapy alone.

Finally, the glucagon-like peptide-1 receptor agonist (GLP-1 RA) class of medications, while initially developed for blood sugar management in patients with diabetes, has demonstrated cardiovascular benefit in both diabetic and non-diabetic patients. Numerous RCTs have shown that semaglutide reduces occurrence of AF by 42% compared to placebo, independent of route of administration [161]. Proposed mechanisms for this include reduction in oxidative stress, inflammation, autonomic nervous system modulation, and reduction in weight-related comorbidities such as hypertension, obstructive sleep apnea, and diabetes [162]. Future research is needed to determine the exact mechanisms through which these medications reduce incident AF, and which patients are most likely to benefit from them.

## 9. Discussion

The French clinical pathologist Jean Baptist de Sénac (1693–1770) was the first to describe a connection between mitral valve stenosis and a “rebellious palpitation” of the heart [163]. Atrial fibrillation, owing to both its long history and its ubiquity, has been well studied in terms of its pathophysiology, treatment, and contribution to other forms of cardiovascular disease. The first described pharmacological treatment for AF was digitalis, derived from foxglove plants, in 1785. Since then, numerous pharmaceutical agents have been developed, chief among them beta-blockers, calcium channel blockers, and the antiarrhythmic class of medications. Historically, most clinical research on AF has focused on comparison of different treatment strategies (i.e., rate vs. rhythm control). In this review, we presented evidence from multiple trials, including RCTs, observational studies, and systematic reviews, comparing the relative efficacy and safety of different agents for the management of AF. While current guidelines are clear under specific circumstances (unstable patients, severe heart failure), most professional societies recommend an adaptive approach; one which takes into consideration patient comorbidities, potential drug–drug interactions, drug cost, and patient preference. There is an abundance of evidence to now suggest that flexibility in AF treatment strategy far outweighs the initial choice of agent, and that, like most medications, individual patient response is impossible to predict and may change over time.

AF management has shifted in recent years from a reactive to a proactive strategy. AF should be managed early and aggressively, especially in younger patients and those with underlying cardiovascular disease and potentially including patients who develop AF in the setting of an acute stressor, as increasing evidence shows this is predictive of recurrence [164]. At the same time, a greater emphasis is now placed on recognition and treatment of underlying risk factors of AF, such as hypertension, obesity, heart failure, and renal disease. New advances in AF detection include pacemakers, implantable loop recorders, and even non-medical wearable technology such as smart watches [165]. With the increasing affordability and portability of diagnostic tools such as handheld ultrasound devices, clinicians practicing in the primary care setting have more opportunities than ever before to screen for early signs of cardiac remodeling associated with paroxysmal AF.

Despite both updated screening guidelines and technological advancements, multiple challenges remain. AF management continues to be limited by medication costs, poor tolerance of pharmaceutical agents (especially in the context of other underlying comorbidities and their respective treatments), and poor healthcare access in general. Beyond this, guidelines for specific situations can be murky. We have outlined, for instance, the lack of definitive recommendations for blood thinners following LAAO device placement, rate versus rhythm control in patients with congenital cardiomyopathies, and choice of anticoagulant for patients with AF in the cancer setting. Similarly, more research is needed to quantify what burden of AF constitutes risk for development of heart disease, and at what stage of detection/management these changes might be reversible. Future progress will likely depend on the study of AF as a continuum of disease, with consistent and frequent monitoring of patient response to their current treatment strategy, as well as a willingness of physicians to make frequent adjustments as patient age and comorbidities evolve.

As a narrative review, this article is not an exhaustive systematic review of all existing literature, but rather, serves as a comprehensive summary of the current guidelines for management of AF, with a focus on RCTs and meta-analyses as evidence supporting these recommendations. This review has several limitations. First, as a narrative review, it focuses mainly on larger-scale studies and may omit smaller clinical trials that examined more niche patient populations. Second, multiple recommendations are based solely on post hoc analyses of trials not originally designed to ask the specific research question addressed by the secondary analysis. While we attempted to include only high-quality evidence, any secondary analysis of existing data is vulnerable to author bias. Finally, heterogeneity across the studies in terms of patient demographics, specific medications examined, and variable endpoints, limit the direct comparability of findings at times. Recommendations provided in this review are therefore based on expert consensus, and, when speculative, this is clearly noted.

## 10. Conclusions

Atrial fibrillation is a complex and incompletely understood disease with multiple independent yet overlapping etiologies. Modern frameworks for both pathogenesis and treatment emphasize recognizing AF as a spectrum of disease rather than a discrete condition. Ultimately, AF does not exist in a vacuum and must be understood in the context of the underlying risk factors that drive it, and its own contribution to other forms of cardiovascular disease. Treatment of AF must therefore balance multiple patient factors, and adequate management should be considered a moving target. As numerous novel therapies emerge, more longitudinal studies will be needed to compare their efficacy and safety at different points in the spectrum of disease.

## Data Availability

No new data were created or analyzed in this study.

## Abbreviations

The following abbreviations are used in this manuscript:

AF	Atrial fibrillation
DOAC	Direct oral anticoagulant
LAAO	Left atrial appendage occlusion
RCT	Randomized controlled trial
HFpEF	Heart Failure with Preserved Ejection Fraction
HFrEF	Heart Failure with Reduced Ejection Fraction
GLP-1 RA	Glucagon-like peptide-1 receptor
PVI	Pulmonary Vein Isolation
